# What is health?

**DOI:** 10.1111/1751-7915.12063

**Published:** 2013-05-06

**Authors:** Harald Brüssow

**Affiliations:** Nestlé Research Center, BioAnalytical Sciences, Food and Health MicrobiologyLausanne, Switzerland

## Abstract

Classical medical research is disease focused and still defines health as absence of disease. Languages, however, associate a positive concept of wholeness with health as does the WHO health definition. Newer medical health definitions emphasize the capacity to adapt to changing external and internal circumstances. The results of the 2010 Global Burden of Disease study provides keys for a quantifiable health metrics by developing statistical tools calculating healthy life expectancy. Of central social and economic importance is the question whether healthy ageing can be achieved. This concept hinges on theories on the biological basis of lifespan determination and whether negligible senescence and the compression of morbidity can be achieved in human societies. Since the health impact of the human gut microbiome is currently a topical research area, microbiologists should be aware of the problems in defining health.

## Introduction

Science has its fashions. Suddenly the leading science journals are full of articles about a specific topical research area. Sometimes, this wave of popularity follows a technological break-through which permits asking questions that were previously impossible to tackle or at least very hard to address experimentally. At other occasions, this cumulating of top-level research reports is the consequence of large international research efforts where grant agencies provided large amounts of money, which attracted many scientists to the field. In still other situations, the scientific community realizes that a certain field of scientific inquiry has simply been overlooked or neglected and the view offered by the new insights is exiting theoretical interest and promising practical applications. The human microbiome is currently such a fashionable field. Novel DNA sequencing techniques combined with new bioinformatic tools and the general progress of ‘–omics’ technologies offer the methods; major research grants on both sides of the Atlantic provided the money and the field has been an eye-opener for microbiologists which might be compared with the time of Leeuwenhoek when microbes in our mouth were first seen in the microscope and the time of Koch when the first isolated bacterial colonies were seen by the naked eye and linked to human disease. We perceive the human microbiome metagenome as our second human genome, as a source of human genetic variability (Schloissnig *et al*., 2013) and as a factor influencing human health (Clemente *et al*., 2012). The human gut microbiome has been associated with health issues of central importance such as obesity (Turnbaugh *et al*., 2006), healthy ageing (Claesson *et al*., 2012) and most recently cancer (Arthur *et al*., 2012), to quote only the most prominent fields. Probiotic bacteria have also been fashionable for a while (Thomas *et al*., 2010) and were judged to have a scientific basis (Neish, 2009), but scientific reports aroused less attention than gut microbiota research. Probiotics carry in their definition as ‘live health-promoting bacteria’ the concept that microbes can influence our health. But what actually is health? If you want to boost health, you must know what it is and how to measure it.

## Health: ask the experts

At school we heard of Socrates who asked people who are supposed to be experts and to get an answer from a dialogue with them. Therefore I first went to health authorities like medical doctors and their authoritative textbooks that guided generations of medical students like Harrison's Principles of Internal Medicine (Longo *et al*., 2011). In the 18th edition you find ample material on pathogens, even a chapter on the human microbiome (Gordon and Knight, 2011), a chapter on women's health, but no definition of health. Overall, one gets the impression that medicine deals with disease and not health. In a recent meeting, one of my colleagues said that the US National Institutes of Health (NIH) should correctly be called National Institutes of Diseases reflecting this disease focus of medical research. Health is currently fashionable as ‘Global Health’, but again scientists working at institutes called like this or in such programmes deal mostly with diseases. After this disappointment, the author turned to PubMed with ‘health’ and ‘definition’ as search terms and got less than 20 papers – a quite surprising outcome for such a central question of the human society. Clearly there is a problem with the definition of the term ‘health’.

## Health: ask the languages

When a term is so self-evident and at the same time so elusive that no definition is provided in the scientific literature, it is frequently helpful to look into the words we use when speaking about it. Naming is the first activity of human beings when trying to make order of things surrounding us. Words reflect the experience of many generations and words constitute a collective subconsciousness that determines still today our unexpressed thoughts and actions, more than we are aware of consciously. In the Oxford Dictionary ‘health’ is defined as ‘the state of being free from illness and injury’. It is obviously a negative definition. Such a definition reflects the current use of the words in the spoken language, but not necessarily its development over time. The English ‘health’ derives from Old English ‘hælth’, which is related to ‘whole’ ‘a thing that is complete in itself’ (Oxford Dictionary) derived from Old English ‘hal’ of Germanic origin (the addition of the w in whole/hal reflects a dialect pronunciation of the 15th century). In Middle English ‘hal’ also became ‘hail’ with the meaning of health in greetings and toasts. ‘hal’ is related to the Dutch ‘heel’ and the German ‘heil’. In German the connections between health, wholeness and salvation becomes even clearer than in English. ‘Heil-kunde’ and ‘Heil-kunst’ are still common German words for medicine, ‘Heiler’ is a traditional or alternative health provider; ‘heilfroh’ means wholly happy, and refers to a relationship between health and happiness. ‘Heil’ has also religious meanings as seen from the German word ‘Heiland’ for the Christ as Saviour (or for false prophets as in ‘Heil Hitler’). The German word conserved clear links with the religious and cultic realm in ‘heilig’ (English: holy) where ‘Heil’ is equivalent with salvation in the religious meaning (‘Seelen-heil’). These connotations are still vibrating consciously or unconsciously in native speakers when using these words. In fact from this quasi-religious context the constitution of the WHO adopted in 1948 becomes understandable when stating ‘the following principles are basic to the happiness, harmonious relations and security of all peoples: Health is a state of complete physical, mental and social wellbeing and not merely the absence of disease or infirmity’. The definition has not been revised, but was variously challenged for its ‘complete wellbeing’ as reflecting a fundamentalist view, referring to an ideal world of messianic expectations. Some scientists have therefore asked for redefining health to make it a realistic, measurable quantity (Saracci, 1997).

Since this language approach turned out to be revealing, let's follow the relationship between health and wellness (are they synonyms or do they express distinct concepts?) and between health and disease (are they antonyms?). Disease is defined by the Oxford Dictionary as ‘disorder of structure or function in an organism that produces specific symptoms and is not the result of physical injury’; ‘dis-ease’ derives from the Old French ‘desaise’ (lack of ease). Wellness and illness is clearly a pair of antonyms. Illr is a Norse word for evil and was taken into Middle English with the meaning of wicked, malevolent. ‘Well’ (German: wohl) derives from a word common to many Germanic languages and means ‘in a good way’, initially as a contrast to wicked. As an adjective one of the meaning of ‘well’ is specifically ‘in good health’ (Oxford Dictionary). In German ‘wohl’ goes beyond good health, it alludes to psychological and emotional aspects (‘Wollust’: English: lust, but in Old English as in current German still in the sense of ‘pleasure’ and ‘delight’) and material wealth (‘Wohlstand’). Wellness thus goes beyond physical health and has a strong connotation of happiness, but also of hedonism (where pleasure is the chief good).

One might argue that these are linguistic associations restricted to Germanic languages. However, this is not the case: the Latin word pair ‘salus’–‘malus’ has very similar connotations which were transmitted into modern Romanic languages (French: salut–maladie). In Latin ‘salus’ means health, rescue, redemption and wealth. It derives from ‘salvus’, Old Indian ‘sarvas’, which meant initially nothing else than ‘whole’. We see here again the notion of completeness with health. Malus which leads then to malady shares with the Germanic word ‘small’ a common root and thus refers to incompleteness. Malus has also moral connotation (Eritis sicut deus scientes bonum et malum – the snake in Genesis: you will be like God knowing the good and the evil). Disease has long been regarded as a celestial punishment for moral failing. In many traditional societies, health surveys should not miss to ask about ‘the evil eye’, underlining the widespread magic concepts on disease.

## Redefining health: medical approaches

Recently the need for a new definition of health was expressed by the *British Medical Journal* (Jadad and O'Grady, 2008). A discussion via global blog conversation was initiated on ‘How should health be defined?’ The participation rate was weak: only 38 communications were counted. In an influential blog, R. Smith (2008) confessed that this issue is for most doctors an uninteresting question since they are interested in disease and not health. Medical textbooks are a massive catalogue of diseases. Health is an illusion and according to the strict standards of the WHO definition, most people are unhealthy for most of the time, so far his comments. Research-oriented doctors complained that the WHO definition has no direct operational value – it is so widely formulated that health outcome cannot easily be measured. Health like beauty is in the eyes of the beholder. It turned out that redefining health is an extremely ambitious and complex goal. A conference held in 2009 in the Netherlands (‘Is health a state or an ability? Towards a dynamic concept of health’) (Huber, 2010), an editorial by the *Lancet* (‘What is health? The ability to adapt’) (Anonymus, 2009) and an analysis in the *BMJ* (‘Health: how should we define it?’) (Huber *et al*., 2011) proposed a few conclusions. The preferred view on health was the ability to adapt and to self-manage. With respect to physical health the term of ‘allostasis’ was introduced – the maintenance of physiological homeostasis through changing circumstances. In the field of mental health a sense of coherence was identified as defining criterion. Social health included people's capacity to fulfil their potentials and obligations, the ability to manage their life and to participate in social activities including work. R. Smith summarized this into the phrase ‘health is the capacity to love and work’ attributed to Sigmund Freud. The Dutch conference highlighted a few important aspects. When applied to ‘successful or healthy ageing’ only a very small percentage of people would fit the WHO definition. When self-rating of well-being was used a much higher percentage rated themselves as successfully ageing and this rating was roughly constant over lifetime. With an ageing population chronic diseases become a life condition to many people. The Stanford Chronic Disease Self-Management Programme uses strategies to enhance self-efficacy which resulted in fewer healthcare requests. Also the WHO has added to this discussion. In preparation of the Ottawa Charter of 1986, the WHO defined health as the ability of an individual to realize aspirations and satisfy needs and to cope with the environment. Health was thus seen as a resource for everyday life. The WHO has also developed an International Classification of Functioning, Disability and Health assessing the performance of a task in real life situation. WHO surveys assessed an individual's health state by asking for mobility, self-care, pain, cognition, interpersonal activities, vision, sleep and energy and affect. The answers go into a single metric reaching from death (0) to perfect health (1). The abovementioned *Lancet* editorial quoted the French physician G. Canguilhem who perceived health in his 1943 book *The Normal and the Pathological* not as something that can be defined statistically or mechanistically. Health is the ability to adapt to one's environment and its own limitations. At the Dutch conference, a participant asked for the concept of ‘salutogenesis’ (becoming healthy) and more concrete research work in a field dominated by studies of pathogenesis (becoming ill). In practical terms it means that instead of carefully observing the conditions that lead from the healthy to the diseased state, research should also be conducted for the opposite process, i.e. the transition from the diseased to the healthy state. In some diseases the transition from health to malady is a way of no return and its inverse process of ‘salutogenesis’ is obviously difficult to study. However, for microbiologists the situation is easier. Many acute infectious diseases show a transition from health to disease followed by a return to the normal. Here ‘salutogenesis’ is commonly studied and had practical outcomes. For example, understanding the immune response to an infectious agent which led to the resolution of the disease was often instrumental for designing vaccine strategies.

## Scaling health levels?

A fundamental question not yet addressed in our discussion is whether health is a state as opposed to the alternative state of disease. There are medical conditions that allow only two alternative conditions; a frequently quoted example is a woman in childbearing age who either is pregnant or is not pregnant. There is no condition where a woman is a bit pregnant, pregnancy is an all-or-nothing event allowing only a ‘plus’ and a ‘minus’ state and no transitions between both of them. At first glance, one might also take ‘health’ and ‘disease’ as alternative ‘plus’ and ‘minus’ states. The self-perception of a subject is a relative reliable measure differentiating a healthy state from a diseased state. In a prodromal phase of an infectious disease, we feel lousy before any overt disease symptoms are evident. During convalescence we feel the reverse process of returning vigour and strength. This distinction finds expression in our outer appearance allowing not only an experienced physician, but even an attentive layperson to differentiate these two states with a single look at a person. This experience speaks for health and disease as two alternative states. However, medical doctors use scoring systems to assess the health and disease status of patients to decide on medical interventions. To quote just two examples: the Karnofsky score runs from 100 (perfect health) to 0 (death) in steps of 10 and assesses the independence or dependence of patients on assistance for everyday activity or survival; its main purpose was to quantify the capacity of cancer patients to cope with chemotherapy. Another score rates the status of newborns: the Apgar score attributes up to two points each for the appearance, pulse, grimace, activity, respiration of the baby (despite this mnemonic, Apgar is named after an anaesthesiologist). Apgar expresses the need for medical intervention by the paediatrician. Apgar scores of 7 or higher characterize healthy babies. These scoring systems are interesting since first, they put health and disease into the same measurable category and second, they anticipate that both health and disease states can be graded. By their design as indicators for medical intervention, these scoring systems have more graded disease levels than graded health levels, but this point can be quickly remedied by introducing a scoring system that depicts in analogy with the number line increasing positive integers to the right as indicators of a graded health level and increasing negative integers to the left as indicators of graded disease levels.

**Figure:**
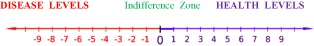


Around 0 is an indifference zone where the subject feels neither particularly healthy nor definitively ill. While numerous scoring systems exist to describe severity grades for many diseases, less scoring systems exist for assessing health levels. This situation could quickly be corrected: Physical strength or mental fitness could be measured quantitatively by performance tests on the subject or functional reserves could be measured by physiological tests on individual organ systems of the subject. Such physical types of test are frequently used in geriatric medicine.

This grading concept – oversimplified as it is – has interesting consequences. When physicians speak about health interventions, they speak mostly about disease interventions where a treatment shifts for example a person from disease level −7 to disease level −3 to remain in the analogy of this fictive scale. Over recent decades medical treatments were also increasingly applied on apparently healthy subjects, who show, but do not suffer, from pathophysiological states (e.g. hypertension, hypercholesterolemia) in order to prevent for example a shift from health level +3 to disease level −7 when the pathophysiological risk factor transforms into actual disease (e.g. myocardial infarct or stroke) (again in this fictive scale). However, physicians and the pharmaceutical industries have much less considered the possibility to increase health levels from for example health state +4 to health state +7 which increases physical and mental performance of the person or the functional reserves of the person's organs. These health interventions were largely left to fitness centres and sport clubs and private activities of the individual. The aim of such nutrition and health interventions would be a better performance in everyday life, more pleasure (quality of life), but not necessarily disease prevention. However, increasing the functional reserve of the body necessarily creates a buffer such that extrinsic factors decreasing the health level do not result that quickly in disease as without this intervention.

## Health: ask the Global Burden of Disease (GBD) 2010 survey

One might argue that health of an individual or a population is to a certain extent a lip service of the medical profession and the true interest of medical doctors is to cure or to prevent disease. Language-wise this focus is expressed by the now frequently used term of ‘ill health’ in the columns of leading journals like ‘Nature’ and ‘The Lancet’, which is of course a clear contradiction in terms and reflects the disease focus of medicine. One might suspect that economists and sociologists have a greater interest in the health of a population when focusing on the productivity and social ‘functioning’ of people. However, such an evaluation does not do justice to the epidemiological, statistical and intellectual efforts of the medical community to come to grip with these terms. The Herculean effort of the medical research field is illustrated by a whole issue of the *Lancet* describing the GBD Study 2010 in a series of articles (Das, 2012). Over 5 years 486 scientists from 302 institutions in 50 countries have collected data on ‘ill health’ and evaluated the data by using the most sophisticated statistical data treatment methods (Murray *et al*., 2012a). The results are stunning. It is here not the place to review these studies, but I want to share with the reader some excitement. From 1970 to 2010 global life expectancy at birth rose by 3–4 years every decade. The resolution of the data set is astonishing: you can for example compare life expectancy per region and per sex. You see then that women in Bangladesh increased their life expectancy from 47.5 years in 1970 to 71.0 years in 2010 (not a printing error). Or you get global life expectancy per 5-year intervals for both sexes, e.g. 80-year-old men had in 1970 a life expectancy of 5.8 years compared with 7.2 years in 2010 (‘the older you get, the healthier you have been’) (Wang *et al*., 2012). Or you get information on 235 leading causes of death separated by age and sex based on files compiling vital registrations, verbal autopsies and various surveillance data from 187 countries. You learn that mortality from communicable diseases has decreased over this time period following major ameliorations in mortality from diarrhoeal diseases, measles and tetanus, but less so for respiratory infections and increases for HIV/AIDS. When the global years of life lost (YLL) is displayed separately for the causes and individual years between 1990 and 2010, the data analysis was so well performed that you see the 1995 famine in North Korea as a sudden increase in global death due to nutritional deficiencies and the 1994 genocide in Rwanda as an intentional injuries increase (Lozano *et al*., 2012).

In the context of our discussion another GBD 2010 report is even more interesting. Salomon and colleagues (2012) start their paper with the statement: ‘Improvement of population health means more than simply delaying death or increasing life expectancy at birth’. They continue: ‘With the trend of population ageing, the need to prioritise healthy ageing is increasingly recognized’. The authors of this paper focus on the description of ‘healthy life expectancy’ as a summary measure of population health. While this term has no particular philosophical or biological foundation, it is based on a lot of sound statistical reasoning. In fact, it goes back on a method developed 40 years ago by D. Sullivan. Healthy life expectancy is the number of years a person at a given age can expect to live in good health taking into account age-specific mortality, morbidity and functional health status. While health is here still largely defined negatively as the absence of disease, it becomes a measurable quantity and thus a simple logically appealing summary measure of population health. The GBD 2010 study goes even further by analysing a composite metric that captures both premature mortality and the prevalence and severity of disease leading to the term of disability-adjusted life years (DALY) (Murray *et al*., 2012b). Health status was measured in other studies by the absence of disability expressed as activity restriction, or absence of dementia, or on a broader basis as a multidimensional expression of functioning. However, with a sufficiently large raw data set one can calculate the ‘healthy life expectancy’ in years. Then the difference between life expectancy minus healthy life expectancy can be interpreted as the average number of years of potentially healthy life lost to poor health. To get back to the above Bangladesh women who had in 2010 a life expectancy of 71 years, they had a healthy life expectancy of 59 years, for Canadian women the two figures were 83 and 68 years respectively. Despite different absolute numbers, women from both countries spent more than a decade with poor health. Interesting trends emerge: both for men and for women global healthy life expectancy has increased by about 4 years between 1990 and 2010 keeping with the overall trend of life expectancy increases. The gains in healthy life expectancy over the past 20 years have mainly been through reductions of child and adult mortality and not through reductions in years lost to disability (YLD). When looking into a study from member states of the European Union, larger variations were found for healthy life expectancy than for life expectancy (Jagger *et al*., 2008). These results are not just about statistics, they represent important elements for political decisions. The UN Millennium Development Goals have focused on the reduction of mortality from major killers like HIV, tuberculosis and malaria. With that focus life expectancy will (hopefully) increase, but it will have minor impact on healthy life expectancy. The computation of healthy life expectancy has changed over the years. Some used dichotomous weighting schemes categorizing people into either healthy or not. The new calculation accounts for the severity of disability calculated for 289 named diseases (Murray *et al*., 2012a) allowing thus a quantitative, gliding disability scale.

## Ageing concepts

The structure of the world population is dramatically changing with an increasing percentage of the human population living to old and very old age (Suzman and Haage, 2011). This phenomenon is not limited to the classical industrialized countries, until 2050 China is expected to reach 440 and 101 million inhabitants older than 60 and 80 years respectively (Shetty, 2012). This change in the population pyramid has not only important socioeconomic consequences (healthcare, pension funds), but affects also the health and disease discussion in an interesting way.

Like for health, everybody knows what ageing means, but definitions are again less obvious and biologists have not yet developed a generally shared theory of ageing (Martin, 2011). Part of the problem might be that different organisms might have their own modes of ageing. Languages are not of much help: ‘age’ is something which can be very simply counted on a timescale. Different languages reflect a different attitude towards ageing: while in English ‘ageing’ implies deterioration, in Japanese it means just the advancement of age. A Japanese researcher has therefore defined ageing as a ‘regression of physiological function accompanied by advancement of age’ (Imahori, 1992). Medical doctors consequently differentiate a chronological and a physiological age of a person.

Medical gerontologists perceive ageing as a progressive decline in structure and function of the body (Ferruci and Studenski, 2011). Most prominent and very visible are the effects of ageing on body composition: lean body mass from muscles and visceral organs decrease steadily, muscle strength decreases (sarcopenia) and is a good predictor of mortality. Progressive demineralization leads to decline of bone strength that together with neurodegeneration induces unstable gait, poor balance and slow reaction times leading to falls and fractures resulting in increasing frailty. Memory decline and dementia are other neurological observations in some, but not all ageing persons. Decline of the sensory system is frequent (vision, hearing, taste). Another physiological change is declining resting metabolic rate with ageing, which is also a marker of illness. Homeostasis pathways (hormones, inflammatory mediators, antioxidants) change progressively with age inducing a lower resistance to stress. Normal ageing is also associated with a decline in food intake particularly in men which leads to malnutrition.

While ageing leads ultimately to death, great biological differences exist for lifespan and ageing process between different organisms. While the lifetime of flies measures in days, some ticks survive for decades and lobsters were reported to survive for more than 100 years without any apparent loss in fertility. Similar data have been reported for turtles, where older females lay more eggs than younger females, show no loss of vigour and no increase in mortality rate with increasing age (Finch, 2009). These observations led to the concept of negligible senescence and the Centenarian Species Project (Guerin, 2004). Negligible senescence contradicts Hamilton's influential theory that natural selection shaped senescence (Hamilton, 1966) and ideas that late survival was sacrificed in evolution for reproduction (Kirkwood and Rose, 1991). Even today, Hamilton's Forces of Natural Selection described in his 1966 paper were compared by evolution researchers to what is the Lorentz transformation for relativistic physics (Rose *et al*., 2007). Of course, working with long-lived animals which might have lifetimes longer than that of the researcher is not to the taste of geneticists who prefer short-lived animals like flies and worms or mostly mice where results are obtained within a grant period. However, negligible senescence would fit other theories, for example that of the French zoologist Buffon who suggested in the 18th century that the duration of life in animals corresponded to six to seven times that of the period of growth for the given animal. An animal which has undetermined growth like some reptiles (crocodiles for example grow as long as they live) could have a very long lifespan. Those zoologists might in fact be right who claim that lobsters die from predation, accident and infection but not as a consequence of ageing.

Many ideas have been developed by biologists on ageing: for example Hayflick developed 40 years ago an argument that the finite number of cell doublings determines the lifespan of a species (Hayflick, 1968). Molecular biologists have added arguments to this idea by highlighting the importance of telomere length shortening with increasing cell divisions. Several other mechanisms and pathways have been revealed by molecular biologists and geneticists for the ageing process. Caloric restriction and longevity is another of the fruitful fields of ageing research. Whether it applies to monkeys as our closest relatives is currently the focus of much discussion (Mattison *et al*., 2012).

However, all what we have discussed so far fit more the fundamental interest of biologists than that of the medical doctor. For the present review let's therefore focus on the human condition and the medical view on healthy ageing.

## Healthy ageing

Thirty years ago Fries (1980) published in *The New England Journal of Medicine* a seminal paper on ‘Ageing, natural death, and the compression of morbidity’ which heavily influenced the medical discussion on ageing. He starts with the statement that the length of life is fixed; speculations on immortality are rooted in human hope. The medical field assumes that death is always the result of a disease process, but due to his hypothesis of a set human lifespan, death might occur without overt disease when the normal span is lived. In his paper he depicted the ‘ideal’ human mortality curve in the absence of premature death: it is a sharp peak around the ‘naturally set’ human lifespan of 85 years. He arrived to this value from the extrapolation of life expectancy data at birth and at age 20 and 65 measured over the last century which intersect in his graph at 85 years. With that idealized model the survival curve of humans has a sharp rectangular form while the actual survival curve for humans at 1900 looked more like a triangle with a continuous decline of survival with age. In 1980 the survival curve took already a substantial rectangular form: much of the 1900-typical attrition over increasing age had been eliminated and the actual survival curve started to approach the ideal curve. He admitted that the average length of life was increasing, but he argues that this was due to a decrease in childhood mortality, not to a secular trend for an increase of life expectancy at age of 75 years. He highlighted that acute, usually infectious diseases determined mortality in the USA at 1900 and that chronic diseases have now superseded acute diseases. In his view health improvement must address chronic instead of acute diseases, morbidity and not mortality, quality of life rather than duration of life. Postponement of disease is more important than cure of a disease. Weight control, regular exercise, treatment of hypertension, elimination of smoking and alcohol over-consumption (today we would add an equilibrated diet) were the practical measures. With that focus of medical interventions, one could achieve what he called the compression of morbidity. A postponement of chronic disease would also result in a rectangularization of the morbidity and not only the mortality curve. Since loss of reserve function represented his operational definition of ageing, one could theoretically also achieve a compression of senescence. He postulated a plasticity of ageing against a non-elasticity of the human ideal lifespan.

It is interesting to compare the Fries' model with the actual data set from the GBD 2010 study. Already in an analysis of demographic data from 2002, the WHO reported that precisely the very old age groups are growing the fastest worldwide. A cornerstone of Fries' model is the lack in increase of centenarians over one century of observation. The WHO projects in contrast a 13-fold increase in centenarians over the next decades (Kalache *et al*., 2002). Better hygiene, nutrition and healthcare have increased life expectancy as also seen in GBD 2010. When the life expectancy of females in the most advanced nations is plotted against historical time, a straight line is observed showing a steady increase of 2.5 years longer life expectancy per decade between 1850 and 2000 (Suzman and Haage, 2011). Humans in some industrialized countries have now nearly reached the lifespan limits of Buffon's formula, but the asymptotic behaviour requested by a genetically fixed life expectancy was not yet observed. One central tenet of the Fries' model is thus not confirmed. What about the compression of morbidity? GBD 2010 showed that countries with high life expectancy had mostly also lower age-specific disability than countries with low life expectancy. While an analysis of disability-adjusted life expectancy (DALE) with data from the GBD 1999 study (Mathers *et al*., 2001) showed still ‘some evidence to suggest that compression of morbidity may be occurring in some low mortality countries’, later analyses did not concur with this interpretation. According to GBD 2010, years lived with disability (YLD) rose despite a decrease in the prevalence of age-specific disability (Salomon *et al*., 2012). Simply, the decrease in disability did not keep pace with the increase in survival. A compression can only occur if healthy life expectancy would rise faster than life expectancy.

Globally, YLD rose from 583 million in 1990 to 777 million in 2010 (Vos *et al*., 2012). The main contributors at the global level were mental and behavioural disorders, musculoskeletal disorders, diabetes and endocrine diseases. The leading specific causes were the same in 2010 as in 1990: low back pain, major depressive disorders, iron-deficiency anaemia, neck pain, chronic obstructive pulmonary disease, anxiety disorders, migraine, diabetes and falls. Rates of YLD per given number of people did not change, but since YLD rise steadily with age, population growth and ageing were the major drivers for the increase in YLD (Vos *et al*., 2012). The health system is thus confronted with a rising number of individuals with a range of disorders that largely cause disability but not mortality.

## Outlook

In summary, GBD 2010 showed clear evidence of expansion, not compression of morbidity. An increase of the number of years lived in reduced health has implications beyond the person suffering from restricted health. Healthy ageing is a socioeconomic need since otherwise national health systems will not be able to stem the cost associated with managing increasing numbers of individuals suffering from various disease sequelae. If by preventive measures a healthy ageing could be achieved, the healthcare system could save cost and the individual could enjoy a greater quality of life for a longer period of life. This goal is quite ambitious though, but the incentive is great justifying the exploration of various associations with healthy ageing. In an accompanying review, I explore the data associating gut microbiota composition with healthy ageing and to what extent the gut microbiota composition can be changed by nutritional interventions (Brüssow, 2013).
